# Evaluation of a Remote Therapeutic Monitoring Device and Integrated Care Platform in Outpatient Parenteral Antimicrobial Therapy

**DOI:** 10.1093/cid/ciag009

**Published:** 2026-01-08

**Authors:** Emily A Siegrist, Mitchell Berenson, Justin D Dvorak, Jacob K Dozier, S Rae Wannier, Rita Wilson Dib, Brian M Scott, Nayle Ibragimova, Debbie Nail Meyer, Maria Alkozah, Michelle Salvaggio, Bryan P White, Bushra Siddique, Sharanjeet Thind, Dale W Bratzler, Joseph Sassine

**Affiliations:** Department of Pharmacy, OU Health, Oklahoma City, Oklahoma, USA; IV Ensure, Inc, Dallas, Texas, USA; Hudson College of Public Health, University of Oklahoma Health Campus, Oklahoma City, Oklahoma, USA; IV Ensure, Inc, Dallas, Texas, USA; IV Ensure, Inc, Dallas, Texas, USA; Division of Infectious Diseases, Department of Medicine, College of Medicine, University of Oklahoma Health Campus, Oklahoma City, Oklahoma, USA; Division of Infectious Diseases, Department of Medicine, College of Medicine, University of Oklahoma Health Campus, Oklahoma City, Oklahoma, USA; Department of Pharmacy, OU Health, Oklahoma City, Oklahoma, USA; IV Ensure, Inc, Dallas, Texas, USA; Division of Infectious Diseases, Department of Medicine, College of Medicine, University of Oklahoma Health Campus, Oklahoma City, Oklahoma, USA; Division of Infectious Diseases, Department of Medicine, College of Medicine, University of Oklahoma Health Campus, Oklahoma City, Oklahoma, USA; Department of Pharmacy, OU Health, Oklahoma City, Oklahoma, USA; Department of Medicine, OU Health Edmond Medical Center, Edmond, Oklahoma, USA; Division of Infectious Diseases, Department of Medicine, College of Medicine, University of Oklahoma Health Campus, Oklahoma City, Oklahoma, USA; Hudson College of Public Health, University of Oklahoma Health Campus, Oklahoma City, Oklahoma, USA; Division of Infectious Diseases, Department of Medicine, College of Medicine, University of Oklahoma Health Campus, Oklahoma City, Oklahoma, USA

**Keywords:** OPAT, adherence, remote therapeutic monitoring

## Abstract

**Background:**

Unplanned healthcare utilization events, including hospital readmissions and emergency room visits, are common in patients receiving outpatient parenteral antimicrobial therapy (OPAT). Little is known about patient adherence to therapy in this setting. This clinical trial evaluates the impact of a remote therapeutic monitoring (RTM) device with an integrated care platform on all-cause healthcare utilization in patients on OPAT.

**Methods:**

This prospective, open-label, nonrandomized study enrolled patients discharged on OPAT to receive the IV Ensure RTM device compared to contemporaneous controls. The primary outcome was all-cause healthcare utilization, including hospital readmission or emergency room visit, assessed at day 90 after discharge. Adherence to antimicrobial therapy was evaluated in the intervention group.

**Results:**

A total of 81 patients were enrolled in the intervention group, with 131 patients in the control group. Both groups had similar baseline and OPAT characteristics. On multivariate Cox regression analysis, the RTM intervention was associated with a 39% reduction in all-cause healthcare utilization by day 60 (adjusted hazard ratio, 0.61 [95% confidence interval, .38–.96]; *P* = .03]) and a 38% reduction by day 90 (0.62 [.40–.95]; *P* = .03). In the intervention group, the overall adherence reached a median of 94%, and 61.7% of patients maintained adherence of >90% throughout their OPAT course.

**Conclusions:**

An RTM device with an integrated care platform significantly reduced all-cause healthcare utilization events in patients on OPAT.

## Teaser

A remote therapeutic monitoring device with an integrated care platform reduced all-cause healthcare utilization (hospital readmissions and emergency room visits) in patients on outpatient parenteral antimicrobial therapy.

## Graphical Abstract

**Graphical Abstract:**
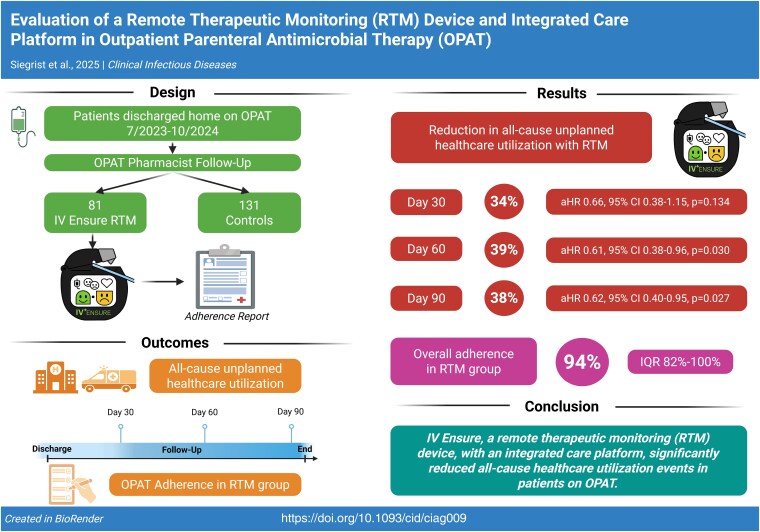
Created in BioRender. Siegrist et al (2025) https://BioRender.com/7wyf1y2

Since the advent of outpatient parenteral antibiotic therapy (OPAT) in the 1970s, OPAT has decreased hospital length of stay and unplanned readmissions, improved patient outcomes and reduced healthcare spending for patients needing prolonged courses of intravenous (antimicrobials but no longer requiring hospitalization [1]. OPAT programs manage outpatient laboratory monitoring, adverse reactions, and central venous catheter complications, and they coordinate care [1, 2]. Many patients receiving OPAT self-administer antimicrobial doses at home. This requires them to be taught about storage, mixing of drugs, administration techniques, and line care [3, 4]. With weekly nurse home visits limited to line care and blood sample collection for safety monitoring, it is difficult to confirm adherence to the administration instructions and schedule without directly observing the administration of medication [4, 5].

Since most OPAT encounters are nonbillable, OPAT programs justify costs based on reduction in unplanned healthcare utilization, typically hospital readmissions [6]. However, readmissions remain common, and studies report rates of 30-day all-cause readmissions between 18% and 27% [7–17]. Risk factors for readmission include discharge to skilled nursing facility [8], age [11], prolonged OPAT duration [7], hematologic cancer [7], aminoglycoside use [9], previous readmissions [7, 9, 11], and endovascular infection [11]. In addition, rates of 30-day all-cause emergency room (ER) visits are between 5% and 10% [18, 19], reaching up to 34% [20].

Given the complexity of intravenous administration for which 38% of patients report no family support [21], we experienced that patients often struggle with self-administration, and data describing validated patient adherence and related medication errors are lacking. These issues are especially true for patients with a complex and time-intensive OPAT regimen [21].

The estimated annual cost of medication nonadherence and nonoptimized treatment in the United States is $528 billion [22]. Electronic monitoring devices have been used with oral and inhaled medications to measure patient adherence [23]. Devices detecting pill bottle opening were associated with decreased viral loads and higher adherence in patients with human immunodeficiency virus [23]; furthermore, devices have been used to improve adherence to inhaled medications for pulmonary diseases [24]. Remote patient management of peritoneal dialysis has used at-home continuous monitoring, improving patient adherence to >90% [25]. In a survey of OPAT patients, 42% of patients expressed firm interest in a monitoring device that reports to their physician, the leading item in a panel of suggestions for improving OPAT [21].

To our knowledge, there are no studies evaluating remote therapeutic monitoring (RTM) for OPAT to identify patients struggling to adhere to the prescribed regimen or to evaluate the impact of RTM on adherence, patient outcomes, and healthcare utilization. This pilot study aims to evaluate the impact of an RTM program on unplanned healthcare utilization (hospital readmissions and ER visits) for patients receiving OPAT. We also aim to describe adherence rates and predictors of adherence.

## METHODS

### Study Design and Patient Population

This was a prospective, open-label, nonrandomized pilot study conducted at 2 hospitals within the University of Oklahoma Health Campus in Oklahoma City, from July 2023 until October 2024. All adult patients discharged home on parenteral antimicrobials were eligible, regardless of OPAT indication or regimen, and were identified from the electronic health record (EHR). Patients discharged to skilled nursing or to acute care facilities and those receiving parenteral antimicrobials at dialysis centers were excluded. Patients who were transitioned to oral antimicrobials after screening but after discharge were also excluded. Patients were approached for consent Monday through Friday, prior to hospital discharge, with the possibility of remote consent should a patient be discharged before being approached. Patients who consented to the intervention and completed an onboarding call the day after discharge were included in the intervention group in an intention-to-treat analysis. The intervention continued until the patients completed their prescribed OPAT course or until they had a hospital readmission event, whichever occurred first. A contemporaneous control group was formed using those who were not enrolled in the intervention group, and data on their outcomes were collected retrospectively from the EHR. The study was approved by the University of Oklahoma Health Campus Institutional Review Board.

### Study Procedures

**Figure 1.**
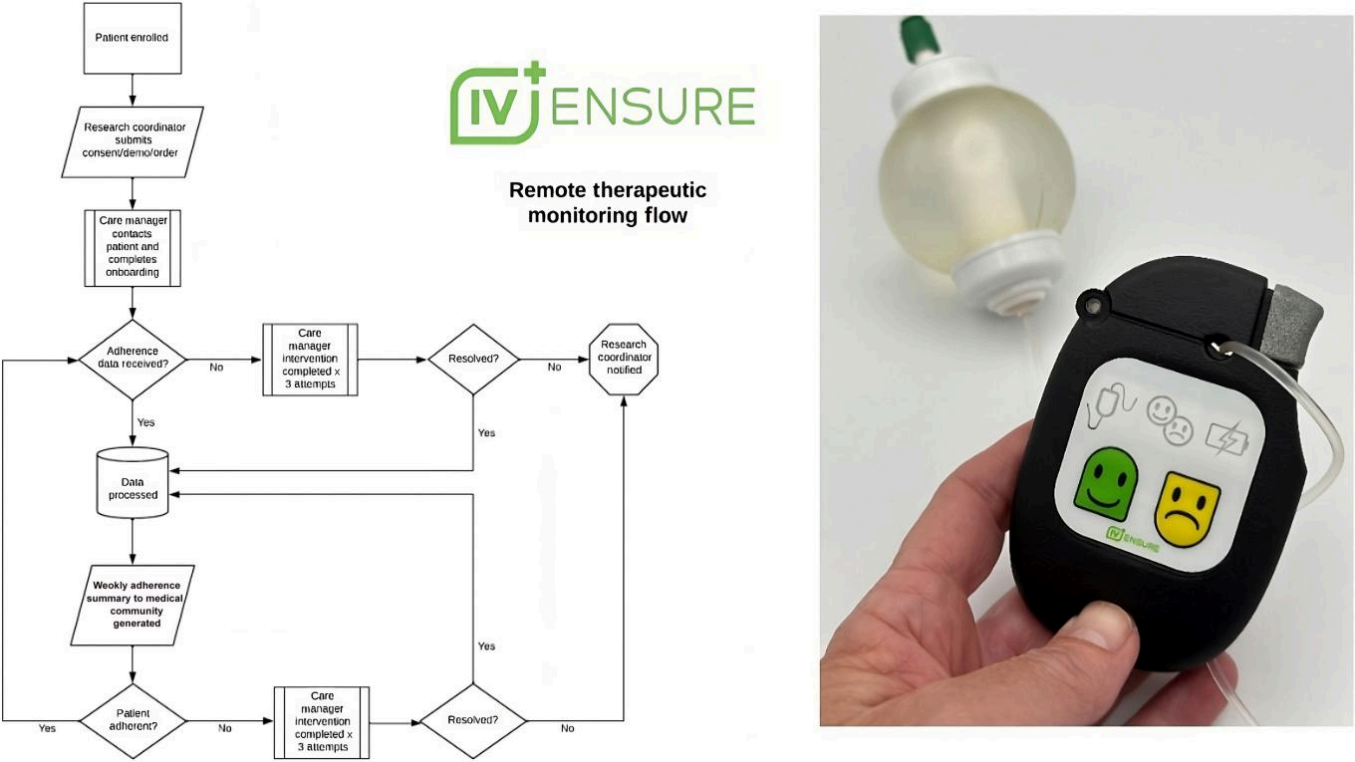
Remote therapeutic monitoring workflow and IV Ensure device. The device attaches directly to the intravenous infusion tubing set and is compatible with any intravenous antimicrobial delivery method. Through proprietary technology, it transmits infusion-related data in real time via cellular connection to IVE Mind, a connected, HITRUST-certified SaaS data repository and care platform. The transmitted data include start and stop times for each infusion and temperature curves, passively captured by IVE Mind to verify actual infusion occurrences. Abbreviation: demo, demonstration.

After consent, patients in the intervention group received the IV Ensure RTM device (Figure 1) at discharge, which they would attach with every infusion. This device is Food and Drug Administration exempt and Medicare approved as a healthcare remote monitoring technology, attaches directly to the intravenous infusion tubing set, and is compatible with any intravenous antimicrobial delivery method (eg, intravenous push, elastomeric pump, or intravenous piggyback). Through proprietary technology, it transmits infusion-related data in real time via cellular connection to IVE Mind, a connected, HITRUST-certified SaaS data repository and care platform.

The transmitted data include start and stop times and infusion temperature curves, passively captured by IVE Mind to verify actual infusion occurrences. These data are compared with the treatment plan prescribed by the treating physician. Adherence is defined as the patient completing the infusion on schedule with the correct daily frequency ≥90% of the time. A weekly adherence report is generated and shared through the EHR.

Each device is paired with an infusion case manager from IV Ensure, who completes an onboarding call with the patient on the day after discharge. Thereafter, IVE Mind's care algorithm queues case managers for required interventions, with daily monitoring of adherence data and weekly follow-up calls until therapy completion (Figure 1). In the event of a missed infusion dose, the patient is first contacted by text message; if ≥2 consecutive medication events are missed, the case manager follows up with a phone call. Patients in the intervention group may reach out to the case manager at any time for support.

The device incorporates an optional mental distress feature through “happy” and “sad” face buttons (Figure 1). If a patient presses the sad face for more than 2 consecutive days, the case manager contacts the patient, as repeated distress signals are a strong indicator of potential therapy nonadherence [26].

Patients in both intervention and control groups were also followed up by the institutional OPAT team as standard of care. This includes a telephone call by the OPAT pharmacist the day after discharge to counsel on medication administration and common adverse events. The OPAT pharmacist reviews weekly laboratory results on all patients, calls patients with significant laboratory abnormalities, and answers patient phone calls and questions [27]. Patients also receive weekly adherence and refill calls from home infusion companies and weekly laboratory monitoring and line care from home health.

### Outcomes

The primary outcome was the proportion of patients with any all-cause healthcare utilization, including hospital readmission or ER visit, whichever occurred first, assessed at days 30, 60, and 90 after discharge. Secondary outcomes included all-cause hospital readmissions and all-cause ER visits, assessed separately at these same time points. Each healthcare utilization event in both groups was reviewed by a blinded adjudicator committee to determine its attribution to one of the following, (1) worsening index infection, (2) new infection at the same site, (3) antimicrobial side effect, (4) vascular access complication, or (5) failure of OPAT setup (definitions in Supplementary Methods). Two adjudicators (B. M. S. and N. I.) blinded to the study group made an initial determination, and a third adjudicator (R.W.D) blinded to the study group and to the first 2 adjudicators' determinations made the decisive determination in case of disagreement (Supplementary Figure 1).

Furthermore, in the intervention group, the overall adherence rate was evaluated as an exploratory outcome, with an aim of identifying predictors of adherence. Low adherence was defined as <90% of doses per week given within 1 hour of the scheduled administration time, and high adherence defined as ≥90%. Adherence was evaluated weekly, and adherence during the first week was compared to that in subsequent weeks of therapy.

### Statistical Analysis

A power analysis estimated that 80 patients are needed in the intervention group to meet a statistical power of 80% for the primary outcome with a prespecified α value of .05, in an intention-to-treat analysis. Categorical variables were compared between groups using χ^2^ or Fisher exact tests as appropriate. Continuous variables were compared using the Mann-Whitney *U* test. Independent predictors of the primary outcome were evaluated by means of Cox regression analysis, with the right-censoring time set at 90 days after discharge. Baseline and OPAT characteristics were individually evaluated in a univariate Cox regression after which, all factors with a *P* <.100, in addition to selected demographic and prognostic variables (age, sex, urban vs rural location, Medicaid insurance, and diabetes mellitus), were incorporated into a multivariate Cox regression analysis with overall significance assessed via likelihood-ratio tests; 95% confidence intervals were estimated using profile likelihood methods. The model was checked for collinearity and for the proportional hazards assumption. Statistical analysis was performed using IBM SPSS software, version 29.0.0.0, and R software, version 4.4.1.

## RESULTS

### Recruitment and Follow-up

**Figure 2.**
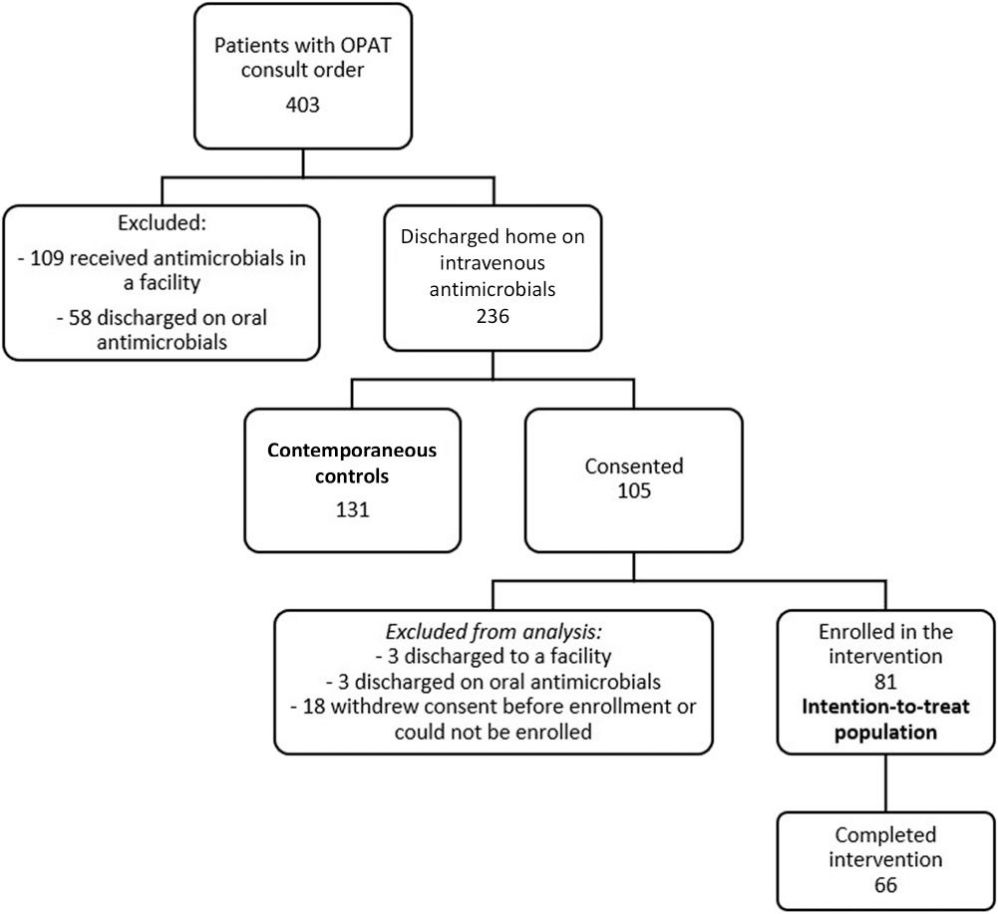
Patient recruitment. Abbreviation: OPAT, outpatient parenteral antimicrobial therapy.

A total of 403 patients had electronic orders for OPAT placed during the study period (Figure 2). Of those, 236 were discharged home on intravenous antimicrobials and were approached for consent; 105 patients consented for participation, and the remaining 131 patients constituted the control group. Of the 105 consenting patients, 81 were enrolled in the intervention group and represented the intention-to-treat population. Fifteen patients (19%) discontinued the intervention prematurely.

### Study Population

Baseline population characteristics were similar between the intervention and control group (Table 1). The most common class of infections was bone and joint (87 patients [41%]), followed by central nervous system/ear, nose and throat (39 [18.4%]) and endovascular infections (37 [17.5%]). The most common comorbid conditions were depression (110 patients [51.9%]) and diabetes mellitus (52 [24.5%]). About a quarter of the patients had received intravenous antibiotics in the preceding year. OPAT characteristics were overall similar between both groups, with a median duration of outpatient therapy of 35 days (interquartile range, 25–39 days) (Table 2). The most commonly used antibiotics were cephalosporins (in 92 patients [43.4%]) and daptomycin (50 [23.6%]); however, more patients received vancomycin in the intervention group (10 [12.3%]) than in the control group (6 [4.6%]; *P* = .04). The number of phone calls conducted by the OPAT team as standard of care was similar in both groups, as were the number of daily antibiotic doses and the total daily infusion times (Table 2).

**Table 1.** Baseline Patient Characteristics by Study Arm

Characteristic	Patients, No. (%)^a^			*P* Value
	Intervention Group (n = 81)	Control Group (n = 131)	Total (N = 212)	
Age, median (IQR), y	57 (44–67)	59 (47–67)	59 (46–67)	.38
Sex				
Female	42 (51.9)	53 (40.5)	95 (44.8)	.10
Male	39 (48.1)	78 (59.5)	117 (55.2)	
Race				
White	66 (81.5)	88 (67.2)	154 (72.6)	.16
African American	7 (8.6)	18 (13.7)	25 (11.8)	
Native American	6 (7.4)	13 (9.9)	19 (9)	
Hispanic	1 (1.2)	9 (6.9)	10 (4.7)	
Asian/Pacific Islander	1 (1.2)	3 (2.3)	4 (1.9)	
Infection class				
Bone and joint	35 (43.2)	52 (39.7)	87 (41)	.39
CNS/ENT	11 (13.6)	28 (21.4)	39 (18.4)	
Endovascular	19 (23.5)	18 (13.7)	37 (17.5)	
Intra-abdominal	9 (11.1)	14 (10.7)	23 (10.8)	
Genitourinary	5 (6.2)	12 (9.2)	17 (8)	
Skin	2 (2.5)	6 (4.6)	8 (3.8)	
Pulmonary	0 (0)	1 (0.8)	1 (0.5)	
Insurance				
Medicare	35 (45.7)	52 (39.7)	89 (42)	.39
Private	20 (24.7)	36 (27.5)	56 (26.4)	.65
Medicaid	20 (24.7)	35 (26.7)	55 (25.9)	.74
VA	3 (3.7)	5 (3.8)	8 (3.8)	>.99
Charity	1 (1.2)	3 (2.3)	4 (1.9)	>.99
Comorbid conditions				
Depression	44 (54.3)	66 (50.4)	110 (51.9)	.58
Diabetes mellitus	19 (23.5)	33 (25.2)	52 (24.5)	.78
Solid tumor cancer	14 (17.3)	25 (19.1)	39 (18.4)	.74
Heart failure	7 (8.6)	10 (7.6)	17 (8)	.79
Solid organ transplant	3 (3.7)	10 (7.6)	13 (6.1)	.38
Hematologic cancer	6 (7.4)	5 (3.8)	11 (5.2)	.34
COPD	2 (2.5)	7 (5.3)	9 (4.2)	.49
End-stage renal disease	2 (2.5)	3 (2.3)	5 (2.4)	>.99
Liver cirrhosis	2 (2.5)	2 (1.5)	4 (1.9)	.64
HCT/CAR T-cell therapy	2 (2.5)	2 (1.5)	4 (1.9)	.64
AIDS	2 (2.5)	0 (0)	2 (.9)	.14
No. of comorbid conditions, median (IQR)	1 (1–2)	1 (1–2)	1 (1–2)	.95
Healthcare utilization in past 12 mo				
Intravenous antibiotics	22 (27.2)	29 (22.1)	51 (24.1)	.40
Intravenous antibiotics for the same type of infection	21 (25.9)	26 (19.8)	47 (22.2)	.33
No. of encounters or admissions, median (IQR)				
Outpatient encounters	5 (2–11)	6 (2–15)	6 (2–13)	.24
ER encounters	1 (0–2)	1 (0–2)	1 (0–2)	.98
Inpatient admissions	1 (0–2)	1 (0–2)	1 (0–2)	.16

Abbreviations: CAR, chimeric antigen receptor; CNS, central nervous system; COPD, chronic obstructive pulmonary disease; ENT, ear nose and throat; ER, emergency room; HCT, hematopoietic cell transplantation; IQR, interquartile range; VA, Veterans Affairs.

^a^Data represent no. (%) of patients unless otherwise specified.

**Table 2.** Outpatient Parenteral Antibiotic Therapy Characteristics by Study Arm

Characteristic	Patients, No. (%)^a^			*P* Value
	Intervention Group (n = 81)	Control Group (n = 131)	Total (N = 212)	
Duration of outpatient therapy, median (IQR), d	35 (25–39)	34 (18–39)	35 (20–39)	.23
No. of phone calls made by OPAT team during OPAT episode, median (IQR)	3 (1–5)	2 (1–4)	2 (1–5)	.86
Antimicrobial class				
Cephalosporins	38 (46.9)	54 (41.2)	92 (43.4)	.42
Daptomycin	19 (23.5)	31 (23.7)	50 (23.6)	.97
Carbapenems	15 (18.5)	25 (19.1)	40 (18.9)	.92
Penicillins	9 (11.1)	26 (19.8)	35 (16.5)	.10
Antifungals	5 (6.2)	17 (13)	22 (10.4)	.11
Metronidazole	7 (8.6)	11 (8.4)	18 (8.5)	.95
Vancomycin	10 (12.3)	6 (4.6)	16 (7.5)	.04
Quinolones	4 (4.9)	3 (2.3)	7 (3.3)	.43
Linezolid	0 (0)	6 (4.6)	6 (2.8)	.08
Antivirals	0 (0)	3 (2.3)	3 (1.4)	.29
TMP/SMX	1 (1.2)	1 (0.8)	2 (0.9)	>.99
Concomitant oral antibiotics	19 (23.5)	36 (27.5)	55 (25.9)	.52
No. of antibiotics, median (IQR)				
Oral	0 (0–0)	0 (0–1)	0 (0–1)	.39
Intravenous	1 (1–1)	1 (1–1)	1 (1–1)	.88
Daily doses of intravenous antibiotics, median (IQR)	2 (1–3)	1 (1–3)	1 (1–3)	.50
Total daily infusion time, median (IQR), min	9 (3–63)	12 (6–183)	9 (6–180)	.10
Vascular access type				
PICC	75 (92.6)	115 (87.8)	190 (89.6)	.53
Midline	2 (2.5)	9 (6.9)	11 (5.2)	
Port	3 (3.7)	6 (4.6)	9 (4.2)	
Other	1 (1.2)	1 (0.8)	2 (0.9)	
Inpatient ID consultation	79 (97.5)	123 (93.9)	202 (95.3)	.11
Outpatient ID clinic follow-up	44 (54.3)	72 (55)	116 (54.7)	.93
Source of laboratory values				
Home health	57 (70.4)	105 (80.2)	162 (76.4)	.059
OU Health infusion clinic	10 (12.3)	17 (13)	27 (12.7)	
Outside laboratory	14 (17.3)	9 (6.9)	23 (10.8)	
MDR infection	24 (29.6)	35 (26.7)	59 (27.8)	.79
MRSA	12 (14.8)	19 (14.5)	31 (14.6)	.95
ESBL GNR	11 (13.6)	10 (7.6)	21 (9.9)	.16
VRE	1 (1.2)	6 (4.6)	7 (3.3)	.26
CRE	0 (0)	1 (0.8)	1 (0.5)	>.99
Diabetic foot infection	12 (14.8)	9 (6.9)	21 (9.9)	.06
Hardware-associated infection	24 (29.6)	42 (32.1)	66 (31.1)	.71
Posttraumatic infection	5 (6.2)	13 (9.9)	18 (8.5)	.34
Discharging service				
Hospital medicine	37 (45.7)	35 (26.8)	71 (34)	.03
Medicine teaching team	17 (21)	38 (29)	55 (25.9)	
Surgery	27 (33.3)	58 (44.3)	85 (40.1)	

Abbreviations: CRE, carbapenem-resistant Enterobacterales; ESBL, extended-spectrum β-lactamase; GNR, gram-negative rod; ID, infectious diseases; MDR, multidrug-resistant; MRSA, methicillin-resistant *Staphylococcus aureus*; OPAT, outpatient parenteral antimicrobial therapy; PICC, peripherally inserted central catheter; TMP/SMX, trimethoprim-sulfamethoxazole; VRE, vancomycin-resistant *Enterococcus*.

^a^Data represent no. (%) of patients unless otherwise specified.

### Primary Outcome

The intervention group experienced lower all-cause healthcare utilization than the control group on the OPAT standard of care (Table 3) at days 30 (28.1% relative reduction; 24.7% vs 34.4%, respectively; *P* = .14), 60 (28.6% relative reduction; 37% vs 51.9%; *P* = .04), and 90 (23.5% relative reduction; 43.2% vs 56.5%; *P* = .06).

**Table 3.** Primary Outcome and Secondary Outcomes by Study Arm

Event	Time After Discharge, d	Event Rate, No. (%)		Unadjusted Analysis	
		Intervention Group (n = 81)	Control Group (n = 131)	Reduction by Intervention, %	*P* Value
All-cause healthcare utilization	30	20 (24.7)	45 (34.4)	28.1	.14
	60	30 (37)	68 (51.9)	28.6	.04
	90	35 (43.2)	74 (56.5)	23.5	.06
Hospital readmission	30	14 (17.3)	28 (21.4)	19.1	.47
	60	21 (25.9)	45 (34.4)	24.5	.20
	90	26 (32.1)	53 (40.5)	20.7	.22
ER visit	30	11 (13.6)	24 (18.3)	25.9	.37
	60	18 (22.2)	35 (26.7)	16.8	.46
	90	20 (24.7)	40 (30.5)	19.1	.36

Abbreviation: ER, emergency room.

In a multivariate Cox regression analysis using a 90-day censoring time, the intervention group had a statistically significant reduction of 39% in the hazard of all-cause healthcare utilization compared with control at day 60 (adjusted hazard ratio, 0.61 [95% confidence interval (CI), .38–.96]; *P* = .03) and a 38% reduction at day 90 (0.62 [.40–.95]; *P* = .03) (Table 4). A similar trend was noted at day 30 (adjusted hazard ratio, 0.66 [95% CI, .38–1.15]; *P* = .13) but did not reach statistical significance (Supplementary Tables 1–2).

**Table 4.** Univariate and Multivariate Cox Regression Analysis of Independent Predictors of All-Cause Healthcare Utilization Events by Day 90

Predictor	Univariate Analysis			Multivariate Analysis		
	HR	95% CI	*P* Value	aHR	95% CI	*P* Value
Study arm						
Control	Reference	…	.051	Reference	…	.03
Intervention	0.67	.45–1.00		0.62	.40–.95	
Age (y)	1.00	.99–1.01	.83	1.00	.99–1.02	.96
Sex						
Female	Reference	…	.36	Reference	…	.31
Male	0.84	.58–1.22		0.80	.53–1.23	
Location						
Urban	Reference	…	.38	Reference	…	.59
Rural	1.31	.72–2.38		1.19	.64–2.21	
Infection class						
Bone and joint	Reference	…	.07	Reference	…	.27
CNS/ENT	1.30	.75–2.25		1.16	.63–2.14	
Endovascular	1.81	1.09–3.03		1.23	.66–2.29	
Intra-abdominal	2.19	1.21–3.99		2.10	1.08–4.08	
Genitourinary	1.43	.71–2.86		0.84	.38–1.83	
Skin	0.55	.13–2.27		0.50	.11–2.18	
Pulmonary	4.51	.61–33.2		2.31	.27–19.9	
Insurance						
Medicaid	1.39	.92–2.09	.12	1.60	.98–2.60	.06
Comorbid conditions						
Diabetes mellitus	1.07	.70–1.63	.77	1.12	.68–1.84	.65
Solid tumor cancer	1.64	1.05–2.57	.04	1.35	.79–2.29	.28
COPD	2.13	1.04–4.39	.06	1.48	.66–3.33	.36
Healthcare utilization in past 12 mo						
No. of ER encounters	1.03	1.01–1.05	.01	1.01	.98–1.04	.63
No. of inpatient admissions	1.12	1.05–1.19	.003	1.09	.99–1.20	.08
No. of phone calls made by OPAT team during OPAT episode	0.90	.84–.97	.004	0.91	.83–.98	.01
Discharging service						
Hospital medicine	Reference	…	.09	Reference	…	.37
Medicine teaching team	1.51	.95–2.42		1.26	.76–2.11	
Surgery	0.91	.58–1.44		0.87	.51–1.46	

Abbreviations: aHR, adjusted hazard ratio; CI, confidence interval; CNS, central nervous system; COPD, chronic obstructive pulmonary disease; ENT, ear nose and throat; ER, emergency room; HR, hazard ratio; OPAT, outpatient parenteral antimicrobial therapy.

### Secondary Outcomes

The intervention group demonstrated trends toward lower all-cause hospital readmissions compared with the control group at days 30, 60, and 90, with reductions ranging between 19.1% and 24.5%, not reaching statistical significance (Table 3). The blinded adjudication process showed a trend toward fewer hospital readmissions due to worsening index infection in the intervention group (4.9% vs 11.5%, respectively; *P* = .14) (Table 5).

**Table 5.** Causes of Healthcare Utilization Events at Day 90, Classified by Study Arm and per Final Blinded Adjudication

Cause	Hospital Readmission			ER Visits		
	Intervention Group, No. (%) (n = 81)	Control Group, No. (%) (n = 131)	*P* Value	Intervention Group, No. (%) (n = 81)	Control Group, No. (%) (n = 131)	*P* Value
Worsening index infection	4 (4.9)	15 (11.5)	.14	1 (1.2)	0 (0)	.38
New infection at same site	4 (4.9)	2 (2.3)	.43	0 (0)	2 (1.5)	.53
Antimicrobial side effect	6 (7.4)	2 (1.5)	.056	0 (0)	0 (0)	>.99
Vascular access complication	1 (1.2)	2 (1.5)	>.99	3 (3.7)	10 (7.6)	.38
Failure of OPAT setup	0 (0)	1 (0.8)	>.99	0 (0)	0 (0)	…

Abbreviations: ER, emergency room; OPAT, outpatient parenteral antimicrobial therapy;

Similar trends toward lower all-cause ER visits at days 30, 60, and 90 were noted in the intervention group compared with the control group, with reduction ranging between 16.8% and 25.9%, not reaching statistical significance (Table 3). The blinded adjudication process revealed a potential signal toward fewer ER visits due to vascular access complications in the intervention group (3.7% vs 7.6%, respectively; *P* = .38) (Table 5). Supplementary Tables 3–5 present the results of the blinded adjudication process.

### Adherence in the Intervention Group

The median overall adherence of individual patients was 94% (interquartile range, 82%–100%). Forty-three patients (53%) required ≥1 additional intervention from the RTM team, with 60% of these interventions occurring within the first week after discharge. The top reasons for additional interventions included patient adherence (33%) and OPAT-related issues (32%) (Table 6). There were 18 patients with an overall adherence rate <80%.

**Table 6.** Remote Therapeutic Monitoring Data in the Intervention Arm

RTM Data	Results in Intervention Arm (n = 81)
Weekly adherence rate, median (IQR), %	
wk 1 (n = 81)	100 (95–100)
wk 2 (n = 74)	100 (94–100)
wk 3 (n = 58)	100 (92–100)
wk 4 (n = 42)	100 (95–100)
wk 5 (n = 32)	99 (91–100)
wk 6 (n = 20)	97 (94–100)
wk 7 (n = 12)	99 (95–100)
wk 8 (n = 6)	98 (94–100)
wk 9 (n = 2)	97 (93–100)
wk 10 (n = 1)	100
wk 11 (n = 1)	93
wk 12 (n = 1)	100
Overall adherence rate, median (IQR)	94 (82–100)
No. of doses, median (IQR)	
Expected	33 (17–78)
Observed	27 (13–60)
Missed	2 (0–9)
Additional intervention from IV Ensure team, no. (%)	43 (53.1)
Within 1st week of OPAT	26/43 (60.4)
Reason for intervention, no. (%)	
Patient adherence	27 (33.3)
OPAT-related issues	26 (32.1)
Unrelated issues	5 (6.2)

Abbreviations: IQR, interquartile range; OPAT, outpatient parenteral antimicrobial therapy; RTM, remote therapeutic monitoring.

We compared patients with an overall adherence rate <90% with those with an overall adherence rate ≥90% (Supplementary Tables 6–8). On multivariate logistic regression, independent predictors of an overall adherence <90% were diabetes mellitus (adjusted odds ratio, 4.81 [95% CI, 1.53–15.1]; *P* = .007) and administration of a penicillin (6.99 [1.48–33.1]; *P* = .01) (Supplementary Table 9).

**Figure 3.**
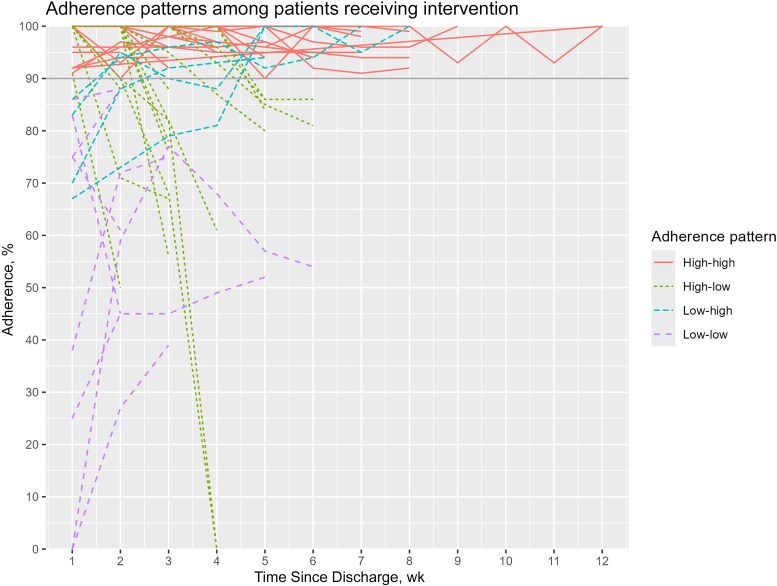
Evolution of weekly adherence over time in the intervention group. This graph represents the 4 patterns of weekly adherence evolution in patients receiving the intervention. High adherence is defined as ≥90%, and low adherence as <90%. Red represents high adherence throughout (50 patients [61.7%]); green, high adherence that drops to <90% (15 patients [18.5%]); gray, low adherence that increases to ≥90% (4 patients [5%]); and blue, low adherence that remains at <90% (12 patients [14.8%]).

Figure 3 illustrates the evolution of weekly adherence over time per patient. Using a cutoff of 90% for high adherence, 4 different patterns emerge, with most patients (50 patients [61.7%]) maintaining high adherence throughout. We compared the patient characteristics in these 4 groups (Supplementary Tables 10 and 11). Patients with persistently low adherence had a median daily infusion time of 80 minutes, significantly higher than in the other groups (median, 6–9 minutes; *P* = .003). All patients with low initial adherence were identified by RTM and received additional support. All patients in the low-high group received additional support in the first OPAT week, which may have resulted in improved adherence thereafter.

## DISCUSSION

In this prospective study of patients discharged on OPAT, an RTM device with an integrated care platform was associated with a statistically significant reduction in all-cause healthcare utilization at days 60 (39%) and 90 (38%) after discharge and a clinically meaningful reduction at day 30 (34%). Overall adherence to the antimicrobial plan reached a median of 94% in the intervention group.

OPAT reduces healthcare utilization by shortening the hospital length of stay and preventing readmissions [6]; however, hospital readmissions and ER visits remain a common occurrence. In our study, RTM was associated with a statistically significant 38% reduction in all-cause healthcare utilization by day 90. We postulate that this reduction was driven by improved adherence and the potential for RTM to identify OPAT-related complications before they lead to a healthcare utilization event, based on the trends noted from the blinded adjudication process, albeit limited by low event rates and subsequent lack of statistical significance. Of note, while clinically meaningful, the reduction in all-cause healthcare utilization at day 30 did not reach statistical significance; 60% of the total cohort and 67% of the intervention group had an outpatient therapy duration of ≥30 days, so the benefits of RTM were more evident at day 60.

The improvements seen in healthcare utilization outcomes may derive from several aspects of the RTM platform. There is the direct impact of the care team being aware and responsive to reports of nonadherence and following up with both the patient and the clinician to address them. The Hawthorne effect also predicts that monitoring adherence directly affects patient behavior and improves adherence [28]. Knowledge of monitoring leads to increased engagement, and individuals tend to become more diligent in taking medications and following care instructions [23]. The magnitude of the effect is linked to the intensity of the monitoring, with more frequent monitoring and more timely feedback eliciting greater improvements in adherence.

The overall adherence, as documented by RTM, was higher than expected, reaching 94%. While adherence had never been objectively measured in OPAT, there are estimates that up to 30% of oral medications are not taken as prescribed [23]. A previous survey of OPAT recipients had self-reported adherence of about 90% [21], but available data show that patients who self-report overestimate their adherence, often by 30%–40% [29–31]. Physicians, likewise, routinely overestimate patient adherence, with nonadherence being equally common in chronic medications and in short-term medications [32–37]. We also observed that >60% of our patients on RTM persisted with high levels of adherence throughout their OPAT course. This higher-than-expected adherence is likely related to a combination of patient commitment, as patients who agreed to enter the trial may have been more adherent, the Hawthorne effect, and the close follow-up through the RTM integrated care platform.

Independent predictors of adherence <90% included diabetes mellitus and receipt of a penicillin, which we commonly administer through a continuous infusion pump. This might reflect patient preference of low time-commitment intravenous pushes as opposed to a 24-hour infusion pump. Patients typically prefer simpler OPAT regimens, exemplified by higher patient satisfaction with daptomycin versus vancomycin [38]. In a survey of 65 OPAT recipients reporting nonadherence rates of 10%, lower adherence was associated with younger age, low household income, lack of support at home, and more frequent antimicrobial dosing [21].

Our study has several strengths. This is the first study to evaluate the use of an adherence monitoring device in patients receiving OPAT and to report an objective measure of adherence to a variety of OPAT regimens. The blinded adjudication process allowed us to better understand factors contributing to healthcare utilization in OPAT. We anticipate that RTM may prove particularly valuable in institutions with smaller or nonexistent OPAT infrastructure. We also believe that RTM will play a major role in OPAT regimens requiring therapeutic drug monitoring, such as vancomycin or aminoglycoside regimens, by providing an accurate record of dose timing.

In terms of limitations, while the intervention and control groups have similar baseline and OPAT characteristics, the study was not randomized and used a contemporaneous control group. Because of this, certain psychosocial differences are unaccounted for, and selection bias could explain the high adherence rates if motivated patients preferentially enrolled in the intervention. In addition, patients in the intervention group received additional calls at discharge and weekly from the RTM team. The reduction in healthcare utilization seen in this study could have been due to improved adherence, increased contact with a care team, or both. Data on readmissions and ER visits were collected retrospectively, and some healthcare utilization events could have been missed if they occurred at outside institutions that do not use Epic.

In conclusion, an RTM device with an integrated care platform significantly reduced all-cause healthcare utilization in patients on OPAT at days 30, 60, and 90 after discharge. Overall adherence reached 94%. Large-scale implementation of RTM will help inform its psychosocial and economic benefits in patients receiving OPAT.

## Supplementary Material

ciag009_Supplementary_Data

## Supplementary Data

Supplementary materials are available at *Clinical Infectious Diseases* online. Consisting of data provided by the authors to benefit the reader, the posted materials are not copyedited and are the sole responsibility of the authors, so questions or comments should be addressed to the corresponding author.

## Notes

***Author contributions.*** Conceptualization: E. A. S., M. B., J. D. D., D. N. M., and J. S. Data curation: E. A. S., R. W. D., B. M. S., N. I., M. A., M. S., B. P. W., and J. S. Formal analysis: J. D. D., S. R. W., and J. S. Funding acquisition and supervision: D. W. B. and J. S. Investigation: E. A. S., J. K. D., B. S., S. T., and J. S. Methodology: M. B., J. D. D., S. R. W., D. N. M., and J. S. Project administration: J. K. D. Validation: R. W. D., B. M. S., N. I., M. A., M. S., and B. P. W. Writing—original draft: E. A. S., M. B., J. D. D., S. R. W., and J. S. Writing—review and editing: J. K. D., N. I., M. A., B. P. W., and D. W. B.

***Acknowledgments.*** The authors thank the study team at the Oklahoma Clinical and Translational Science Institute, including Brittany Karfonta, Sukrutha Thotala, Blair Apple Hill, and Gina Herbert, as well as Quincey Krein from the OU Health Edmond Medical Center, for supporting this study.

***Data availability statement.*** Data are available from the corresponding author upon reasonable request.

***Financial support.*** This work was supported by Community Infusion Solutions and IV Ensure (grant to the University of Oklahoma; principal investigator, J. S.). The study personnel were supported by the Oklahoma Shared Clinical and Translational Resources (U54GM104938) with an Institutional Development Award (IDeA) from NIGMS.
