# Supplementary material for: Evaluation of a Remote Therapeutic Monitoring Device and Integrated Care Platform in Outpatient Parenteral Antimicrobial Therapy
Source: Clin Infect Dis. 2026 Jan 8;82(5):e899–909. doi: 10.1093/cid/ciag009 (PMC13189677; doi:10.1093/cid/ciag009)
Supplement: ciag009_Supplementary_Data [file ciag009_supplementary_data.docx]

**Supplement to:** **Evaluation of a Remote Therapeutic Monitoring (RTM) Device and Integrated Care Platform in Outpatient Parenteral Antimicrobial Therapy (OPAT)**

**Supplemental Methods**

*Outcomes*

The below definitions were provided to the blinded adjudicators to determine the cause of each healthcare utilization event:

When evaluating a healthcare utilization event, please consider the two following criteria:

1. Unplanned (excludes planned admissions for chemotherapy or staged surgeries for example) AND
2. Primary cause of the event as determined by the time of discharge.

Evaluate for the following as causes of healthcare utilization events:

1. Worsening index infection at the same site – Needs to meet both criteria 1 and 2:
   - This includes patients who initially responded to therapy but then worsened before achieving full resolution, or patients who never responded to therapy.
   1. The worsening infection at the same site is diagnosed based on clinical findings (signs and symptoms based on a physician’s note), radiographic findings (findings on appropriate imaging), or microbiological findings (such as a persistently positive culture or PCR) which are worsening or which have never improved compared to the index admission, and which are available by the time the patient discharged.
   2. The worsening infection has resulted in a change in antimicrobial therapy or an extension in antimicrobial therapy or in additional source control procedures during the event.
2. New infection at the same site of the index infection – Needs to meet both criteria:
3. Needs clear documentation of clinical resolution (based on a physician’s note) or of radiographic resolution (based on appropriate interval imaging) or of microbiological resolution (based on a negative microbiological test such as culture or PCR) before the new infection is diagnosed.
4. The new infection is diagnosed based on new clinical findings (new signs and symptoms based on a physician’s note), new radiographic findings (new findings on appropriate imaging), or new microbiological findings (such as a newly positive culture or PCR) that are available by the time the patient discharged.
5. Antimicrobial side effect – Needs to meet all of the following 3 criteria:
6. A known side effect of the antimicrobial.
7. Determined to be related to the antimicrobial by the time the patient discharged.
8. Resulted in discontinuation of the antimicrobial.
9. Vascular access complication (line infection, clot, malfunction, line accidentally pulled, needing line care).
10. Failure of OPAT setup.

**Supplemental Figure 1 – Blinded Adjudication Process Workflow**

**Supplemental Results**

**Supplemental Table 1 – Univariate & Multivariate Cox Regression Analysis of Independent Predictors of All-Cause Healthcare Utilization Events by Day 30**

| **Predictor** | **Univariate** | | | **Multivariate** | | |
| --- | --- | --- | --- | --- | --- | --- |
|  | **HR** | **95% CI** | **p-value** | **aHR** | **95% CI** | **p-value** |
| *Study arm* |  |  |  |  |  |  |
| Control | Ref. |  |  | Ref. |  |  |
| Intervention | 0.67 | 0.39-1.13 | 0.122 | 0.66 | 0.38-1.15 | 0.134 |
| Age (years) | 1.00 | 0.98-1.01 | 0.730 | 1.00 | 0.98-1.02 | 0.759 |
| *Sex* |  |  |  |  |  |  |
| Female | Ref. |  |  | Ref. |  |  |
| Male | 0.62 | 0.38-1.02 | 0.057 | 0.80 | 0.53-1.23 | 0.103 |
| *Location* |  |  | 0.723 |  |  |  |
| Urban | Ref. |  |  | Ref. |  |  |
| Rural | 1.44 | 0.68-3.08 | 0.350 | 1.55 | 0.71-3.40 | 0.278 |
| *Infection class* |  |  | 0.029 |  |  | 0.071 |
| Bone and joint | Ref. |  |  | Ref. |  |  |
| CNS/ENT | 2.18 | 1.09-4.37 |  | 1.69 | 0.78-3.68 |  |
| Endovascular | 2.07 | 1.02-4.21 |  | 1.14 | 0.50-2.64 |  |
| Intra-abdominal | 3.56 | 1.70-7.46 |  | 2.72 | 1.16-6.36 |  |
| Genitourinary | 1.19 | 0.40-3.53 |  | 0.49 | 0.14-1.66 |  |
| Skin | 1.27 | 0.29-5.49 |  | 0.98 | 0.21-4.59 |  |
| Pulmonary | 5.65 | 0.75-42.6 |  | 2.34 | 0.24-22.5 |  |
| *Insurance* |  |  |  |  |  |  |
| Medicaid | 1.54 | 0.92-2.57 | 0.111 | 1.72 | 0.92-3.22 | 0.093 |
| *Comorbidities* |  |  |  |  |  |  |
| Diabetes mellitus | 0.61 | 0.32-1.17 | 0.120 | 0.78 | 0.37-1.66 | 0.514 |
| Solid tumor malignancy | 2.21 | 1.30-3.74 | 0.006 | 1.55 | 0.82-2.94 | 0.182 |
| Chronic obstructive pulmonary disease | 1.18 | 0.37-3.77 | 0.781 | 1.03 | 0.29-3.71 | 0.963 |
| *Healthcare utilization in the past 12 months* |  |  |  |  |  |  |
| Number of ER encounters | 1.03 | 1.01-1.05 | 0.063 | 1.01 | 0.97-1.06 | 0.559 |
| Number of inpatient admissions | 1.12 | 1.05-1.19 | 0.003 | 1.04 | 0.91-1.20 | 0.549 |
| Number of phone calls done by OPAT team during OPAT episode | 1.08 | 0.99-1.18 | 0.138 | 0.88 | 0.78-0.99 | 0.023 |
| *Discharging service* |  |  | 0.145 |  |  | 0.304 |
| Hospital medicine | Ref. |  |  | Ref. |  |  |
| Medicine teaching team | 1.51 | 0.84-2.73 |  | 1.36 | 0.71-2.60 |  |
| Surgery | 0.84 | 0.46-1.54 |  | 0.81 | 0.42-1.58 |  |

*Abbreviations – CNS, central nervous system; ENT, ear nose and throat; ER, emergency room; OPAT, outpatient parenteral antimicrobial therapy.*

**Supplemental Table 2 – Univariate & Multivariate Cox Regression Analysis of Independent Predictors of All-Cause Healthcare Utilization Events by Day 60**

| **Predictor** | **Univariate** | | | **Multivariate** | | |
| --- | --- | --- | --- | --- | --- | --- |
|  | **HR** | **95% CI** | **p-value** | **aHR** | **95% CI** | **p-value** |
| *Study arm* |  |  |  |  |  |  |
| Control | Ref. |  |  | Ref. |  |  |
| Intervention | 0.64 | 0.41-0.98 | 0.034 | 0.61 | 0.38-0.96 | 0.030 |
| Age (years) | 1.00 | 0.99-1.01 | 0.962 | 1.00 | 0.99-1.02 | 0.791 |
| *Sex* |  |  |  |  |  |  |
| Female | Ref. |  |  | Ref. |  |  |
| Male | 0.83 | 0.56-1.24 | 0.378 | 0.84 | 0.54-1.30 | 0.426 |
| *Location* |  |  |  |  |  |  |
| Urban | Ref. |  |  | Ref. |  |  |
| Rural | 1.20 | 0.64-2.27 | 0.567 | 1.12 | 0.58-2.13 | 0.742 |
| *Infection class* |  |  | 0.073 |  |  | 0.273 |
| Bone and joint | Ref. |  |  | Ref. |  |  |
| CNS/ENT | 1.54 | 0.87-2.72 |  | 1.25 | 0.67-2.36 |  |
| Endovascular | 1.84 | 1.06-3.19 |  | 1.25 | 0.64-2.42 |  |
| Intra-abdominal | 2.41 | 1.29-4.53 |  | 2.22 | 1.10-4.48 |  |
| Genitourinary | 1.48 | 0.70-3.09 |  | 0.83 | 0.37-1.90 |  |
| Skin | 0.67 | 0.16-2.78 |  | 0.56 | 0.13-2.49 |  |
| Pulmonary | 4.77 | 0.65-35.2 |  | 2.30 | 0.26-20.2 |  |
| *Insurance* |  |  |  |  |  |  |
| Medicaid | 1.46 | 0.95-2.24 | 0.089 | 1.70 | 1.02-2.82 | 0.044 |
| *Comorbidities* |  |  |  |  |  |  |
| Diabetes mellitus | 0.90 | 0.56-1.43 | 0.644 | 0.95 | 0.56-1.64 | 0.864 |
| Solid tumor malignancy | 1.75 | 1.11-2.78 | 0.023 | 1.40 | 0.81-2.44 | 0.238 |
| Chronic obstructive pulmonary disease | 1.96 | 0.91-4.22 | 0.119 | 1.59 | 0.67-3.78 | 0.311 |
| *Healthcare utilization in the past 12 months* |  |  |  |  |  |  |
| Number of ER encounters | 1.03 | 1.01-1.05 | 0.043 | 1.02 | 0.98-1.06 | 0.310 |
| Number of inpatient admissions | 1.07 | 0.99-1.16 | 0.106 | 1.02 | 0.92-1.15 | 0.683 |
| Number of phone calls done by OPAT team during OPAT episode | 0.89 | 0.82-0.96 | 0.002 | 0.90 | 0.82-0.98 | 0.013 |
| *Discharging service* |  |  | 0.102 |  |  | 0.335 |
| Hospital medicine | Ref. |  |  | Ref. |  |  |
| Medicine teaching team | 1.54 | 0.94-2.52 |  | 1.37 | 0.80-2.33 |  |
| Surgery | 0.93 | 0.57-1.51 |  | 0.93 | 0.53-1.60 |  |

*Abbreviations – CNS, central nervous system; ENT, ear nose and throat; ER, emergency room; OPAT, outpatient parenteral antimicrobial therapy.*

**Supplemental Table 3 –** **Causes of Hospital Readmission Events, Classified by Study Arm and Source of Adjudication**

| **Cause** | **Blinded Adjudicator 1** | | **Blinded Adjudicator 2** | | **Final Blinded Adjudication** | |
| --- | --- | --- | --- | --- | --- | --- |
|  | **Intervention**  **(n=81)** | **Control**  **(n=131)** | **Intervention**  **(n=81)** | **Control**  **(n=131)** | **Intervention**  **(n=81)** | **Control**  **(n=131)** |
| Worsening index infection | 4 (4.9%) | 16 (12.2%) | 6 (7.4%) | 14 (10.7%) | 4 (4.9%) | 15 (11.5%) |
| New infection at the same site | 3 (3.7%) | 1 (0.8%) | 2 (2.5%) | 4 (3.1%) | 4 (4.9%) | 2 (2.3%) |
| Antimicrobial side effect | 5 (6.2%) | 2 (1.5%) | 6 (7.4%) | 2 (1.5%) | 6 (7.4%) | 2 (1.5%) |
| Vascular access complication | 1 (1.2%) | 2 (1.5%) | 0 (0%) | 1 (0.8%) | 1 (1.2%) | 2 (1.5%) |
| Failure of OPAT setup | 0 (0%) | 1 (0.8%) | 0 (0%) | 2 (1.5%) | 0 (0%) | 1 (0.8%) |

*Overall inter-rater agreement was 86%, with a Cohen’s kappa of 0.72.*

*Abbreviations – OPAT, outpatient parenteral antimicrobial therapy.*

**Supplemental Table 4 – Causes of ER Visit Events, Classified by Study Arm and Source of Adjudication**

| **Cause** | **Blinded Adjudicator 1** | | **Blinded Adjudicator 2** | | **Final Blinded Adjudication** | |
| --- | --- | --- | --- | --- | --- | --- |
|  | **Intervention**  **(n=81)** | **Control**  **(n=131)** | **Intervention**  **(n=81)** | **Control**  **(n=131)** | **Intervention**  **(n=81)** | **Control**  **(n=131)** |
| Worsening index infection | 0 (0%) | 1 (0.8%) | 1 (1.2%) | 0 (0%) | 1 (1.2%) | 0 (0%) |
| New infection at the same site | 0 (0%) | 2 (1.5%) | 0 (0%) | 2 (1.5%) | 0 (0%) | 2 (1.5%) |
| Antimicrobial side effect | 0 (0%) | 0 (0%) | 0 (0%) | 0 (0%) | 0 (0%) | 0 (0%) |
| Vascular access complication | 3 (3.7%) | 10 (7.6%) | 4 (4.9%) | 10 (7.6%) | 3 (3.7%) | 10 (7.6%) |
| Failure of OPAT setup | 0 (0%) | 0 (0%) | 0 (0%) | 0 (0%) | 0 (0%) | 0 (0%) |

*Overall inter-rater agreement was 95%, with a Cohen’s kappa of 0.87.*

*Abbreviations – ER, emergency room; OPAT, outpatient parenteral antimicrobial therapy.*

**Supplemental Table 5 – Discordant Adjudications Between the First Two Blinded Adjudicators and Final Adjudication Outcome**

| **Type of Event** | **Study Arm** | **Blinded Adjudicator 1** | **Blinded Adjudicator 2** | **Blinded Adjudicator 3** | **Final Blinded Adjudication** |
| --- | --- | --- | --- | --- | --- |
| Readmission | Control | None | Worsening index infection | Worsening index infection | Worsening index infection |
| Readmission | Control | None | Failure of OPAT setup | None | None |
| Readmission | Control | Worsening index infection | None | Worsening index infection | Worsening index infection |
| Readmission | Control | None | New infection at the same site | None | None |
| Readmission | Control | Vascular access complication | None | Vascular access complication | Vascular access complication |
| Readmission | Control | Worsening index infection | None | Worsening index infection | Worsening index infection |
| Readmission | Control | None | Worsening index infection | None | None |
| Readmission | Intervention | None | Worsening index infection | Worsening index infection | Worsening index infection |
| Readmission | Intervention | Vascular access complication | None | Vascular access complication | Vascular access complication |
| Readmission | Intervention | None | Worsening index infection | None | None |
| Readmission | Intervention | New infection at the same site | None | New infection at the same site | New infection at the same site |
| ER visit | Control | Worsening index infection | None | None | None |
| ER visit | Intervention | None | Worsening index infection | Worsening index infection | Worsening index infection |
| ER visit | Intervention | None | Vascular access complication | None | None |

*Abbreviations – ER, emergency room; OPAT, outpatient parenteral antimicrobial therapy.*

**Supplemental Table 6 – Baseline Characteristics of Intervention Patients by Overall Adherence**

| **Characteristic** | **Adherence**  **< 90%**  **(n=28)** | **Adherence**  **≥ 90%**  **(n=53)** | **Total Intervention**  **(n=81)** | **p-value** |
| --- | --- | --- | --- | --- |
| Age (years) | 58 (37-69) | 57 (45-64) | 57 (44-67) | 0.905 |
| *Sex* |  |  |  | 0.488 |
| Female | 16 (57.1%) | 26 (49.1%) | 42 (51.9%) |  |
| Male | 12 (42.9%) | 27 (50.9%) | 39 (48.1%) |  |
| *Race* |  |  |  | 0.674 |
| White | 25 (89.3%) | 41 (77.4%) | 66 (81.5%) |  |
| African American | 2 (7.1%) | 5 (9.4%) | 7 (8.6%) |  |
| Native American | 1 (3.6%) | 5 (9.4%) | 6 (7.4%) |  |
| Hispanic | 0 (0%) | 1 (1.9%) | 1 (1.2%) |  |
| Asian/Pacific Islander | 0 (0%) | 1 (1.9%) | 1 (1.2%) |  |
| *Infection class* |  |  |  | 0.169 |
| Bone and joint | 12 (42.9%) | 23 (43.4%) | 35 (43.2%) |  |
| CNS/ENT | 5 (17.9%) | 6 (11.3%) | 11 (13.6%) |  |
| Endovascular | 3 (10.7%) | 16 (30.2%) | 19 (23.5%) |  |
| Intra-abdominal | 5 (17.9%) | 4 (7.5%) | 9 (11.1%) |  |
| Genitourinary | 3 (10.7%) | 2 (3.8%) | 5 (6.2%) |  |
| Skin | 0 (0%) | 2 (3.8%) | 2 (2.5%) |  |
| *Insurance* |  |  |  |  |
| Medicare | 14 (50%) | 23 (43.4%) | 37 (45.7%) | 0.570 |
| Private | 6 (21.4%) | 14 (26.4%) | 20 (24.7%) | 0.621 |
| Medicaid | 7 (25%) | 13 (24.5%) | 20 (24.7%) | 0.963 |
| VA | 1 (3.6%) | 2 (3.8%) | 3 (3.7%) | 1.000 |
| Charity | 0 (0%) | 1 (1.9%) | 1 (1.2%) | 1.000 |
| *Comorbidities* |  |  |  |  |
| Depression | 16 (57.1%) | 28 (52.8%) | 44 (54.3%) | 0.711 |
| Diabetes mellitus | 11 (39.3%) | 8 (15.1%) | 19 (23.5%) | 0.015 |
| Solid tumor malignancy | 6 (21.4%) | 8 (15.1%) | 14 (17.3%) | 0.542 |
| Heart failure | 2 (7.1%) | 5 (9.4%) | 7 (8.6%) | 1.000 |
| Hematological malignancy | 0 (0%) | 6 (11.3%) | 6 (7.4%) | 0.088 |
| Solid organ transplant | 1 (3.6%) | 2 (3.8%) | 3 (3.7%) | 1.000 |
| Chronic obstructive pulmonary disease | 2 (7.1%) | 0 (0%) | 2 (2.5%) | 0.117 |
| End stage renal disease | 1 (3.6%) | 1 (1.9%) | 2 (2.5%) | 1.000 |
| Liver cirrhosis | 0 (0%) | 2 (3.8%) | 2 (2.5%) | 0.542 |
| HCT/CAR T cell therapy | 0 (0%) | 2 (3.8%) | 2 (2.5%) | 0.542 |
| AIDS | 1 (3.6%) | 1 (1.9%) | 2 (2.5%) | 1.000 |
| *Healthcare utilization in the past 12 months* | | | | |
| IV antibiotics | 9 (32.1%) | 13 (24.5%) | 22 (27.2%) | 0.464 |
| IV antibiotics for an infection of the same type | 9 (32.1%) | 12 (22.6%) | 21 (25.9%) | 0.353 |
| Number of outpatient encounters | 6 (2-12) | 5 (1-11) | 5 (2-11) | 0.988 |
| Number of ER encounters | 0 (0-3) | 1 (0-2) | 1 (0-2) | 0.664 |
| Number of inpatient admissions | 1 (0-2) | 1 (0-2) | 1 (0-2) | 0.729 |

*Abbreviations – CNS, central nervous system; ENT, ear nose and throat; VA, Veterans Affairs; HCT, hematopoietic cell transplantation; CAR T cell, chimeric antigen receptor T cell; AIDS, acquired immunodeficiency syndrome; IV, intravenous; ER, emergency room.*

*Categorical variables are presented as number (percentage), continuous variables as median (IQR).*

**Supplemental Table 7 – OPAT Characteristics of Intervention Patients by Overall Adherence**

| **Characteristic** | **Adherence**  **< 90%**  **(n=28)** | **Adherence**  **≥ 90%**  **(n=53)** | **Total Intervention**  **(n=81)** | **p-value** |
| --- | --- | --- | --- | --- |
| Outpatient days of therapy (days) | 36 (24-40) | 34 (25-38) | 35 (25-39) | 0.673 |
| Number of phone calls done by OPAT team during OPAT episode | 2 (0-5) | 3 (2-5) | 3 (1-5) | 0.169 |
| *Antimicrobial class* |  |  |  |  |
| Cephalosporins | 11 (39.3%) | 27 (50.9%) | 38 (46.9%) | 0.317 |
| Daptomycin | 8 (28.6%) | 11 (20.8%) | 19 (23.5%) | 0.430 |
| Carbapenems | 6 (21.4%) | 9 (17%) | 15 (18.5%) | 0.624 |
| Vancomycin | 4 (14.3%) | 6 (11.3%) | 10 (12.3%) | 0.731 |
| Penicillins | 6 (21.4%) | 3 (5.7%) | 9 (11.1%) | 0.058 |
| Metronidazole | 2 (7.1%) | 5 (9.4%) | 7 (8.6%) | 1.000 |
| Antifungals | 2 (7.1%) | 3 (5.7%) | 5 (6.2%) | 1.000 |
| Quinolones | 0 (0%) | 4 (7.5%) | 4 (4.9%) | 0.293 |
| TMP/SMX | 0 (0%) | 1 (1.9%) | 1 (1.2%) | 1.000 |
| Concomitant oral antibiotics | 7 (25%) | 12 (22.6%) | 19 (23.5%) | 0.812 |
| Number of oral antibiotics | 0 (0-1) | 0 (0) | 0 (0) | 0.850 |
| Number of IV antibiotics | 1 (1-1) | 1 (1-1) | 1 (1-1) | 0.073 |
| Number of daily doses of IV antibiotics | 2 (1-3) | 2 (1-3) | 2 (1-3) | 0.885 |
| Total daily infusion time (minutes) | 30 (8-105) | 9 (3-30) | 9 (3-63) | 0.106 |
| *Vascular access type* |  |  |  | 0.117 |
| PICC | 26 (92.9%) | 49 (92.5%) | 75 (92.6%) |  |
| Midline | 2 (7.1%) | 0 (0%) | 2 (2.5%) |  |
| Port | 0 (0%) | 3 (5.7%) | 3 (3.7%) |  |
| Other | 0 (0%) | 1 (1.9%) | 1 (1.2%) |  |
| Inpatient ID consult | 27 (96.4%) | 52 (98.1%) | 79 (97.5%) | 0.297 |
| Outpatient ID clinic follow up | 18 (64.3%) | 26 (49.1%) | 44 (54.3%) | 0.191 |
| *Labs obtained through* |  |  |  | 0.584 |
| Home health | 21 (75%) | 36 (67.9%) | 57 (70.4%) |  |
| OU infusion clinic | 2 (7.1%) | 8 (15.1%) | 10 (12.3%) |  |
| Outside lab | 5 (17.9%) | 9 (17%) | 14 (17.3%) |  |
| Multidrug resistant infection | 9 (32.1%) | 15 (28.3%) | 24 (29.6%) | 0.719 |
| MRSA | 5 (17.9%) | 7 (13.2%) | 12 (14.8%) | 0.743 |
| ESBL GNR | 4 (14.3%) | 7 (13.2%) | 11 (13.6%) | 1.000 |
| VRE | 0 (0%) | 1 (1.9%) | 1 (1.2%) | 1.000 |
| Diabetic foot infection | 7 (25%) | 5 (9.4%) | 12 (14.8%) | 0.097 |
| Hardware-associated infection | 8 (28.6%) | 16 (30.2%) | 24 (29.6%) | 0.880 |
| Post-traumatic infection | 0 (0%) | 5 (9.4%) | 5 (6.2%) | 0.158 |
| *Discharging service* |  |  |  | 0.074 |
| Hospital medicine | 16 (57.1%) | 21 (39.6%) | 37 (45.7%) |  |
| Medicine teaching team | 2 (7.1%) | 15 (28.3%) | 17 (21%) |  |
| Surgery | 10 (35.7%) | 17 (32.1%) | 27 (33.3%) |  |

*Abbreviations – OPAT, outpatient parenteral antimicrobial therapy; TMP/SMX, trimethoprim-sulfamethoxazole; IV, intravenous; PICC, peripherally inserted central catheter; ESBL, extended-spectrum beta-lactamase; GNR, Gram-negative rod; VRE, vancomycin-resistant Enterococcus; MRSA, methicillin-resistant Staphylococcus aureus; CRE, carbapenem-resistant Enterobacterales.*

*Categorical variables are presented as number (percentage), continuous variables as median (IQR).*

**Supplemental Table 8 – Remote Therapeutic Monitoring & Outcomes of Intervention Patients by Overall Adherence**

| **Characteristic** | **Adherence**  **< 90%**  **(n=28)** | **Adherence**  **≥ 90%**  **(n=53)** | **Total Intervention**  **(n=81)** | **p-value** |
| --- | --- | --- | --- | --- |
| *Adherence Pattern* |  |  |  | < 0.001 |
| High-High | 2 (7.1%) | 48 (90.6%) | 50 (61.7%) |  |
| High-Low | 12 (42.9%) | 3 (5.7%) | 15 (18.5%) |  |
| Low-High | 2 (7.1%) | 2 (3.8%) | 4 (4.9%) |  |
| Low-Low | 12 (42.9%) | 0 (0%) | 12 (14.8%) |  |
| Overall adherence rate | 66 (40-82) | 100 (94-100) | 94 (81-100) | < 0.001 |
| Number of doses expected | 36 (25-89) | 28 (15-71) | 33 (17-78) | 0.093 |
| Number of doses observed | 26 (11-49) | 27 (14-68) | 27 (13-60) | 0.235 |
| Number of doses missed | 13 (8-24) | 0 (0-2) | 2 (0-9) | < 0.001 |
| Additional intervention from IVEnsure team | 25 (89.3%) | 18 (34%) | 43 (53.1%) | < 0.001 |
| Within the first week of OPAT | 17 (60.7%) | 9 (17%) | 26 (32.1%) | < 0.001 |
| *Reason for intervention* |  |  |  |  |
| Patient adherence | 20 (70.4%) | 7 (13.2%) | 27 (33.3%) | < 0.001 |
| OPAT-related issues | 13 (46.2%) | 13 (24.5%) | 26 (32.1%) | 0.045 |
| Unrelated issues | 3 (10.7%) | 2 (3.8%) | 5 (6.2%) | 0.334 |
| *All-cause healthcare utilization events* | | | | |
| 30 days | 5 (17.9%) | 15 (28.3%) | 20 (24.7%) | 0.300 |
| 60 days | 7 (25%) | 23 (43.4%) | 30 (37%) | 0.103 |
| 90 days | 9 (32.1%) | 26 (49.1%) | 35 (43.2%) | 0.144 |
| *All-cause readmission* |  |  |  |  |
| 30 days | 5 (17.9%) | 9 (17%) | 14 (17.3%) | 1.000 |
| 60 days | 5 (17.9%) | 16 (30.2%) | 21 (25.9%) | 0.228 |
| 90 days | 7 (25%) | 19 (35.8%) | 26 (32.1%) | 0.320 |
| *Adjudicated cause of readmission* | | | | |
| Worsening index infection | 1 (3.6%) | 3 (5.7%) | 4 (4.9%) | 1.000 |
| New infection at the same site | 1 (3.6%) | 3 (5.7%) | 4 (4.9%) | 1.000 |
| Antimicrobial side effect | 3 (10.7%) | 3 (5.7%) | 6 (7.4%) | 0.411 |
| Vascular access complication | 1 (3.6%) | 0 (0%) | 1 (1.2%) | 0.346 |
| *All-cause ER visit* |  |  |  |  |
| 30 days | 3 (10.7%) | 8 (15.1%) | 11 (13.6%) | 0.740 |
| 60 days | 5 (17.9%) | 13 (24.5%) | 18 (22.2%) | 0.492 |
| 90 days | 6 (21.4%) | 14 (26.4%) | 20 (24.7%) | 0.621 |
| *Adjudicated cause of ER visit* | | | | |
| Worsening index infection | 0 (0%) | 1 (1.9%) | 1 (1.2%) | 1.000 |
| Vascular access complication | 0 (0%) | 3 (5.7%) | 3 (3.7%) | 0.548 |

*Abbreviations – OPAT, outpatient parenteral antimicrobial therapy; ER, emergency room.*

*Categorical variables are presented as number (percentage), continuous variables as median (IQR).*

**Supplemental Table 9 – Predictors of Overall Adherence < 90% in the Intervention Group**

| **Predictor** | **Univariate** | | | **Multivariate** | | |
| --- | --- | --- | --- | --- | --- | --- |
|  | **OR** | **95% CI** | **p-value** | **aOR** | **95% CI** | **p-value** |
| Age (years) | 1.00 | 0.97-1.03 | 0.950 |  |  |  |
| *Sex* |  |  |  |  |  |  |
| Female | Ref. |  |  |  |  |  |
| Male | 0.72 | 0.29-1.82 | 0.489 |  |  |  |
| *Race* |  |  |  |  |  |  |
| White | Ref. |  |  |  |  |  |
| African American | 0.66 | 0.12-3.64 | 0.630 |  |  |  |
| Native American | 0.33 | 0.04-2.97 | 0.322 |  |  |  |
| *Infection class* |  |  |  |  |  |  |
| Bone and joint | Ref. |  |  |  |  |  |
| CNS/ENT | 1.60 | 0.40-6.33 | 0.505 |  |  |  |
| Endovascular | 0.36 | 0.09-1.48 | 0.157 |  |  |  |
| Intra-abdominal | 2.40 | 0.54-10.6 | 0.250 |  |  |  |
| Genitourinary | 2.88 | 0.42-19.6 | 0.281 |  |  |  |
| *Insurance* |  |  |  |  |  |  |
| Medicare | 1.30 | 0.52-3.27 | 0.571 |  |  |  |
| Private | 0.76 | 0.26-2.26 | 0.621 |  |  |  |
| Medicaid | 1.03 | 0.36-2.96 | 0.963 |  |  |  |
| VA | 0.94 | 0.08-10.9 | 0.963 |  |  |  |
| *Comorbidities* |  |  |  |  |  |  |
| Depression | 1.19 | 0.47-3.00 | 0.711 |  |  |  |
| Diabetes mellitus | 3.64 | 1.25-10.6 | 0.018 | 4.81 | 1.53-15.1 | 0.007 |
| Solid tumor malignancy | 1.53 | 0.47-4.97 | 0.475 |  |  |  |
| Heart failure | 0.74 | 0.13-4.07 | 0.728 |  |  |  |
| Solid organ transplant | 0.94 | 0.08-10.9 | 0.963 |  |  |  |
| End stage renal disease | 1.93 | 0.12-32.0 | 0.648 |  |  |  |
| *Healthcare utilization in the past 12 months* |  |  |  |  |  |  |
| IV antibiotics | 1.46 | 0.53-4.00 | 0.465 |  |  |  |
| IV antibiotics for an infection of the same type | 1.62 | 0.58-4.49 | 0.355 |  |  |  |
| Number of outpatient encounters | 1.00 | 0.96-1.05 | 0.907 |  |  |  |
| Number of ER encounters | 1.07 | 0.89-1.28 | 0.468 |  |  |  |
| Number of inpatient admissions | 1.02 | 0.87-1.19 | 0.801 |  |  |  |
| Outpatient days of therapy (days) | 1.00 | 0.97-1.03 | 0.859 |  |  |  |
| Number of phone calls done by OPAT team during OPAT episode | 0.93 | 0.80-1.09 | 0.381 |  |  |  |
| *Antimicrobial class* |  |  |  |  |  |  |
| Cephalosporins | 0.62 | 0.25-1.58 | 0.319 |  |  |  |
| Daptomycin | 1.53 | 0.53-4.39 | 0.431 |  |  |  |
| Carbapenems | 1.33 | 0.42-4.22 | 0.625 |  |  |  |
| Penicillins | 4.54 | 1.04-19.8 | 0.044 | 6.99 | 1.48-33.1 | 0.014 |
| Antifungals | 1.28 | 0.20-8.16 | 0.792 |  |  |  |
| Metronidazole | 0.74 | 0.13-4.07 | 0.728 |  |  |  |
| Vancomycin | 1.31 | 0.34-5.07 | 0.700 |  |  |  |
| Concomitant oral antibiotics | 1.14 | 0.39-3.32 | 0.812 |  |  |  |
| Number of oral antibiotics | 1.02 | 0.38-2.77 | 0.965 |  |  |  |
| Number of IV antibiotics | 3.34 | 0.86-13.0 | 0.082 | 2.38 | 0.54-10.5 | 0.253 |
| Number of daily doses of IV antibiotics | 0.95 | 0.62-1.47 | 0.825 |  |  |  |
| Total daily infusion time (minutes) | 1.00 | 0.99-1.00 | 0.414 |  |  |  |
| Outpatient ID clinic follow up | 1.87 | 0.73-4.79 | 0.193 |  |  |  |
| *Labs obtained through* |  |  | 0.598 |  |  |  |
| Home health | Ref. |  |  |  |  |  |
| OU infusion clinic | 0.43 | 0.08-2.21 | 0.311 |  |  |  |
| Outside lab | 0.95 | 0.28-3.22 | 0.937 |  |  |  |
| Multidrug resistant infection | 1.20 | 0.44-3.24 | 0.719 |  |  |  |
| MRSA | 1.43 | 0.41-5.00 | 0.577 |  |  |  |
| ESBL GNR | 1.10 | 0.29-4.12 | 0.893 |  |  |  |
| Diabetic foot infection | 3.20 | 0.91-11.2 | 0.070 |  |  |  |
| Hardware-associated infection | 0.92 | 0.34-2.54 | 0.880 |  |  |  |
| *Discharging service* |  |  | 0.106 |  |  |  |
| Hospital medicine | Ref. |  |  |  |  |  |
| Medicine teaching team | 0.18 | 0.04-0.88 | 0.034 |  |  |  |
| Surgery | 0.77 | 0.28-2.13 | 0.618 |  |  |  |

*Abbreviations – CNS, central nervous system; ENT, ear nose and throat; VA, Veterans Affairs; IV, intravenous; ER, emergency room; OPAT, outpatient parenteral antimicrobial therapy; PICC, peripherally inserted central catheter; ESBL, extended-spectrum beta-lactamase; GNR, Gram-negative rod; MRSA, methicillin-resistant Staphylococcus aureus.*

*Multivariate model included all variables with p < 0.100 on univariate model. Diabetic foot infection excluded from the multivariate model due to collinearity with diabetes mellitus.*

**Supplemental Table 10 – Baseline and OPAT Characteristics of Patients in the Intervention Group According to Adherence Patterns**

| **Characteristic** | **High-High**  **(n=50)** | **High-Low**  **(n=15)** | **Low-High**  **(n=4)** | **Low-Low**  **(n=12)** | **Total Intervention**  **(n=81)** | **p-value** |
| --- | --- | --- | --- | --- | --- | --- |
| Age (years) | 57 (45-62) | 63 (40-72) | 53 (34-63) | 56 (31-72) | 57 (44-67) | 0.608 |
| *Sex* |  |  |  |  |  | 0.411 |
| Female | 23 (46%) | 10 (66.7%) | 3 (75%) | 6 (50%) | 42 (51.9%) |  |
| Male | 27 (54%) | 5 (33.3%) | 1 (25%) | 6 (50%) | 39 (48.1%) |  |
| *Race* |  |  |  |  |  | 0.984 |
| White | 38 (76%) | 13 (86.7%) | 4 (100%) | 11 (91.7%) | 66 (81.5%) |  |
| African American | 5 (10%) | 1 (6.7%) | 0 (0%) | 1 (8.3%) | 7 (8.6%) |  |
| Native American | 5 (10%) | 1 (6.7%) | 0 (0%) | 0 (0%) | 6 (7.4%) |  |
| Hispanic | 1 (2%) | 0 (0%) | 0 (0%) | 0 (0%) | 1 (1.2%) |  |
| Asian/Pacific Islander | 1 (2%) | 0 (0%) | 0 (0%) | 0 (0%) | 1 (1.2%) |  |
| *Infection class* |  |  |  |  |  | 0.179 |
| Bone and joint | 20 (40%) | 9 (60%) | 3 (75%) | 3 (25%) | 35 (43.2%) |  |
| CNS/ENT | 8 (16%) | 1 (6.7%) | 0 (0%) | 2 (16.7%) | 11 (13.6%) |  |
| Endovascular | 15 (30%) | 2 (13.3%) | 0 (0%) | 2 (16.7%) | 19 (23.5%) |  |
| Intra-abdominal | 4 (8%) | 1 (6.7%) | 0 (0%) | 4 (33.3%) | 9 (11.1%) |  |
| Genitourinary | 1 (2%) | 2 (13.3%) | 1 (25%) | 1 (8.3%) | 5 (6.2%) |  |
| Skin | 2 (4%) | 0 (0%) | 0 (0%) | 0 (0%) | 2 (2.5%) |  |
| *Insurance* |  |  |  |  |  |  |
| Medicare | 19 (38%) | 10 (66.7%) | 1 (25%) | 7 (58.3%) | 37 (45.7%) | 0.150 |
| Private | 14 (28%) | 2 (13.3%) | 2 (50%) | 2 (16.7%) | 20 (24.7%) | 0.372 |
| Medicaid | 14 (28%) | 3 (20%) | 1 (25%) | 2 (16.7%) | 20 (24.7%) | 0.828 |
| VA | 2 (4%) | 0 (0%) | 0 (0%) | 1 (8.3%) | 3 (3.7%) | 0.691 |
| Charity | 1 (2%) | 0 (0%) | 0 (0%) | 0 (0%) | 1 (1.2%) | 0.890 |
| *Comorbidities* |  |  |  |  |  |  |
| Depression | 28 (56%) | 9 (60%) | 1 (25%) | 6 (50%) | 44 (54.3%) | 0.631 |
| Diabetes mellitus | 8 (16%) | 7 (46.7%) | 1 (25%) | 3 (25%) | 19 (23.5%) | 0.108 |
| Solid tumor malignancy | 7 (14%) | 2 (13.3%) | 0 (0%) | 5 (41.7%) | 14 (17.3%) | 0.095 |
| Heart failure | 5 (10%) | 1 (6.7%) | 0 (0%) | 1 (8.3%) | 7 (8.6%) | 0.903 |
| Solid organ transplant | 2 (4%) | 1 (6.7%) | 0 (0%) | 0 (0%) | 3 (3.7%) | 0.802 |
| Hematological malignancy | 6 (12%) | 0 (0%) | 0 (0%) | 0 (0%) | 6 (7.4%) | 0.260 |
| Chronic obstructive pulmonary disease | 0 (0%) | 1 (6.7%) | 0 (0%) | 1 (8.3%) | 2 (2.5%) | 0.243 |
| End stage renal disease | 1 (2%) | 1 (6.7%) | 0 (0%) | 0 (0%) | 2 (2.5%) | 0.671 |
| Liver cirrhosis | 2 (4%) | 0 (0%) | 0 (0%) | 0 (0%) | 2 (2.5%) | 0.736 |
| HCT/CAR T cell therapy | 2 (4%) | 0 (0%) | 0 (0%) | 0 (0%) | 2 (2.5%) | 0.736 |
| *Healthcare utilization in the past 12 months* |  |  |  |  |  |  |
| IV antibiotics | 11 (22%) | 5 (33.3%) | 2 (50%) | 4 (33.3%) | 22 (27.2%) | 0.523 |
| IV antibiotics for an infection of the same type | 10 (20%) | 5 (33.3%) | 2 (50%) | 4 (33.3%) | 21 (25.9%) | 0.408 |
| Number of outpatient encounters | 5 (1-11) | 7 (3-11) | 4 (2-7) | 11 (3-16) | 5 (2-11) | 0.421 |
| Number of ER encounters | 1 (0-2) | 1 (0-3) | 0 (0-1) | 0 (0-5) | 1 (0-2) | 0.612 |
| Number of inpatient admissions | 1 (0-2) | 1 (0-2) | 1 (1-2) | 1 (1-3) | 1 (0-2) | 0.658 |
| Outpatient days of therapy (days) | 34 (23-39) | 37 (32-40) | 36 (24-38) | 35 (24-39) | 35 (25-39) | 0.642 |
| Number of phone calls done by OPAT team during OPAT episode | 3 (2-5) | 2 (0-5) | 8 (2-13) | 1 (0-6) | 3 (1-5) | 0.332 |
| *Antimicrobial class* |  |  |  |  |  |  |
| Cephalosporins | 23 (46%) | 9 (60%) | 3 (75%) | 3 (25%) | 38 (46.9%) | 0.201 |
| Daptomycin | 10 (20%) | 6 (40%) | 1 (25%) | 2 (16.7%) | 19 (23.5%) | 0.402 |
| Carbapenems | 9 (18%) | 2 (13.3%) | 0 (0%) | 4 (33.3%) | 15 (18.5%) | 0.402 |
| Penicillins | 5 (10%) | 1 (6.7%) | 0 (0%) | 3 (25%) | 9 (11.1%) | 0.361 |
| Antifungals | 3 (6%) | 0 (0%) | 0 (0%) | 2 (16.7%) | 5 (6.2%) | 0.316 |
| Metronidazole | 5 (10%) | 1 (6.7%) | 0 (0%) | 1 (8.3%) | 7 (8.6%) | 0.903 |
| Vancomycin | 6 (12%) | 1 (6.7%) | 0 (0%) | 3 (25%) | 10 (12.3%) | 0.425 |
| Quinolones | 3 (6%) | 1 (6.7%) | 0 (0%) | 0 (0%) | 4 (4.9%) | 0.790 |
| TMP/SMX | 1 (2%) | 0 (0%) | 0 (0%) | 0 (0%) | 1 (1.2%) | 0.890 |
| Concomitant oral antibiotics | 11 (22%) | 3 (20%) | 0 (0%) | 5 (41.7%) | 19 (23.5%) | 0.308 |
| Number of oral antibiotics | 0 (0) | 0 (0) | 0 (0) | 0 (0-1) | 0 (0) | 0.331 |
| Number of IV antibiotics | 1 (1-1) | 1 (1-1) | 1 (1-1) | 1 (1-2) | 1 (1-1) | 0.100 |
| Number of daily doses of IV antibiotics | 2 (1-3) | 1 (1-3) | 2 (1-3) | 3 (2-3) | 2 (1-3) | 0.608 |
| Total daily infusion time (minutes) | 9 (3-30) | 9 (3-12) | 6 (3-95) | 80 (45-152) | 9 (3-63) | 0.003 |
| *Vascular access type* |  |  |  |  |  | 0.247 |
| Midline | 0 (0%) | 2 (13.3%) | 0 (0%) | 0 (0%) | 2 (2.5%) |  |
| PICC | 46 (92%) | 13 (86.7%) | 4 (100%) | 12 (100%) | 75 (92.6%) |  |
| Port | 3 (6%) | 0 (0%) | 0 (0%) | 0 (0%) | 3 (3.7%) |  |
| Other | 1 (2%) | 0 (0%) | 0 (0%) | 0 (0%) | 1 (1.2%) |  |
| Inpatient ID consult | 49 (98%) | 14 (93.3%) | 4 (100%) | 12 (100%) | 79 (97.5%) | 0.536 |
| Outpatient ID clinic follow up | 23 (46%) | 11 (73.3%) | 3 (75%) | 7 (48.3%) | 44 (54.3%) | 0.226 |
| *Labs obtained through* |  |  |  |  |  | 0.149 |
| Home health | 32 (64%) | 11 (73.3%) | 2 (50%) | 12 (100%) | 57 (70.4%) |  |
| OU infusion clinic | 8 (16%) | 2 (13.3%) | 0 (0%) | 0 (0%) | 10 (12.3%) |  |
| Outside lab | 10 (20%) | 2 (13.3%) | 2 (50%) | 0 (0%) | 14 (17.3%) |  |
| Multidrug resistant infection | 14 (28%) | 4 (26.7%) | 2 (50%) | 4 (33.3%) | 24 (29.6%) | 0.801 |
| MRSA | 6 (12%) | 2 (13.3%) | 2 (50%) | 2 (16.7%) | 12 (14.8%) | 0.231 |
| ESBL GNR | 7 (14%) | 2 (13.3%) | 0 (0%) | 2 (16.7%) | 11 (13.6%) | 0.865 |
| VRE | 1 (2%) | 0 (0%) | 0 (0%) | 0 (0%) | 1 (1.2%) | 0.890 |
| Diabetic foot infection | 5 (10%) | 5 (33.3%) | 1 (25%) | 1 (8.3%) | 12 (14.8%) | 0.126 |
| Hardware-associated infection | 13 (26%) | 5 (33.3%) | 2 (50%) | 4 (33.3%) | 24 (29.6%) | 0.732 |
| Post-traumatic infection | 5 (10%) | 0 (0%) | 0 (0%) | 0 (0%) | 5 (6.2%) | 0.347 |
| *Discharging service* |  |  |  |  |  | 0.028 |
| Hospital medicine | 18 (36%) | 8 (53.3%) | 4 (100%) | 7 (58.3%) | 37 (45.7%) |  |
| Medicine teaching team | 16 (32%) | 0 (0%) | 0 (0%) | 1 (8.3%) | 17 (21%) |  |
| Surgery | 16 (32%) | 7 (46.7%) | 0 (0%) | 4 (33.3%) | 27 (33.3%) |  |

*Abbreviations – CNS, central nervous system; ENT, ear nose and throat; VA, Veterans Affairs; HCT, hematopoietic cell transplantation; CAR T cell, chimeric antigen receptor T cell; IV, intravenous; ER, emergency room; OPAT, outpatient parenteral antimicrobial therapy; TMP/SMX, trimethoprim-sulfamethoxazole; PICC, peripherally inserted central catheter; ESBL, extended-spectrum beta-lactamase; GNR, Gram-negative rod; VRE, vancomycin-resistant Enterococcus; MRSA, methicillin-resistant Staphylococcus aureus; CRE, carbapenem-resistant Enterobacterales.*

*Categorical variables are presented as number (percentage), continuous variables as median (IQR).*

**Supplemental Table 11 -** **Outcomes of Patients in the Intervention Group by Adherence Patterns**

| **Characteristic** | **High-High**  **(n=50)** | **High-Low**  **(n=15)** | **Low-High**  **(n=4)** | **Low-Low**  **(n=12)** | **Total Intervention**  **(n=81)** | **p-value** |
| --- | --- | --- | --- | --- | --- | --- |
| Overall adherence rate | 100 (95-100) | 80 (50-86) | 91 (86-95) | 43 (28-68) | 94 (82-100) | < 0.001 |
| Number of doses expected | 28 (14-61) | 42 (26-87) | 97 (93-112) | 34 (19-37) | 33 (17-78) | 0.020 |
| Number of doses observed | 27 (13-60) | 27 (14-68) | 86 (83-103) | 9 (7-28) | 27 (13-60) | 0.002 |
| Number of doses missed | 0 (0-2) | 13 (4-17) | 9 (6-14) | 13 (8-30) | 2 (0-9) | < 0.001 |
| Additional intervention from IVEnsure team | 15 (30%) | 12 (80%) | 4 (100%) | 12 (100%) | 43 (53.1%) | < 0.001 |
| Within the first week of OPAT | 6 (12%) | 6 (40%) | 4 (100%) | 10 (83.3%) | 26 (32.1%) | < 0.001 |
| *Reason for intervention* |  |  |  |  |  |  |
| Patient adherence | 6 (12%) | 9 (60%) | 2 (50%) | 10 (83.3%) | 27 (33.3%) | < 0.001 |
| OPAT-related issues | 10 (50%) | 5 (33.3%) | 4 (100%) | 7 (58.3%) | 26 (32.1%) | 0.001 |
| Unrelated issues | 0 (0%) | 4 (26.7%) | 0 (0%) | 1 (8.3%) | 5 (6.2%) | 0.002 |
| *All-cause healthcare utilization events* | | | | | | |
| 30 days | 14 (28%) | 2 (13.3%) | 1 (25%) | 3 (25%) | 20 (24.7%) | 0.721 |
| 60 days | 21 (42%) | 3 (20%) | 2 (50%) | 4 (33.3%) | 30 (37%) | 0.431 |
| 90 days | 24 (48%) | 5 (33.3%) | 2 (50%) | 4 (33.3%) | 35 (43.2%) | 0.656 |
| *All-cause readmission* |  |  |  |  |  |  |
| 30 days | 8 (16%) | 2 (13.3%) | 1 (25%) | 3 (25%) | 14 (17.3%) | 0.828 |
| 60 days | 15 (30%) | 2 (13.3%) | 1 (25%) | 3 (25%) | 21 (25.9%) | 0.642 |
| 90 days | 18 (36%) | 4 (26.7%) | 1 (25%) | 3 (25%) | 26 (32.1%) | 0.820 |
| *Adjudicated cause of readmission* | | | | | | |
| Worsening index infection | 3 (6%) | 0 (0%) | 1 (25%) | 0 (0%) | 4 (4.9%) | 0.175 |
| New infection at the same site | 3 (6%) | 1 (6.7%) | 0 (0%) | 0 (0%) | 4 (4.9%) | 0.790 |
| Antimicrobial side effect | 2 (4%) | 2 (13.3%) | 0 (0%) | 2 (16.7%) | 6 (7.4%) | 0.329 |
| Vascular access complication | 0 (0%) | 0 (0%) | 0 (0%) | 1 (8.3%) | 1 (1.2%) | 0.121 |
| *All-cause ER visit* |  |  |  |  |  |  |
| 30 days | 8 (16%) | 1 (6.7%) | 1 (25%) | 1 (8.3%) | 11 (13.6%) | 0.662 |
| 60 days | 12 (24%) | 2 (13.3%) | 2 (50%) | 2 (16.7%) | 18 (22.2%) | 0.427 |
| 90 days | 13 (26%) | 3 (20%) | 2 (50%) | 2 (16.7%) | 20 (24.7%) | 0.569 |
| *Adjudicated cause of ER visit* | | | | | | |
| Worsening index infection | 1 (2%) | 0 (0%) | 0 (0%) | 0 (0%) | 1 (1.2%) | 0.890 |
| Vascular access complication | 3 (6%) | 0 (0%) | 0 (0%) | 0 (0%) | 3 (3.7%) | 0.587 |

*Abbreviations – OPAT, outpatient parenteral antimicrobial therapy; ER, emergency room.*

*Categorical variables are presented as number (percentage), continuous variables as median (IQR).*
